# Production of itaconate by whole-cell bioconversion of citrate mediated by expression of multiple cis-aconitate decarboxylase (*cadA*) genes in *Escherichia coli*

**DOI:** 10.1038/srep39768

**Published:** 2017-01-04

**Authors:** Junyoung Kim, Hyung-Min Seo, Shashi Kant Bhatia, Hun-Seok Song, Jung-Ho Kim, Jong-Min Jeon, Kwon-Young Choi, Wooseong Kim, Jeong-Jun Yoon, Yun-Gon Kim, Yung-Hun Yang

**Affiliations:** 1 Department of Biological Engineering, College of Engineering, Konkuk University, 1 Hwayang-dong, Gwangjin-gu, Seoul, 143-701, Republic of Korea; 2 Institute for Ubiquitous Information Technology and Applications (CBRU), Konkuk University, Seoul 143-701, Republic of Korea; 3 Department of Environmental Engineering, Ajou University, 206, World cup-ro, Yeongtong-gu, Suwon, Gyeonggi-do, 443-749, Republic of Korea; 4 Division of Infectious Diseases, Rhode Island Hospital, Alpert Medical School of Brown University, Providence, Rhode Island, United States of America; 5 IT Convergence Materials R&BD Group, Chungcheong Regional Division Korea Institute of Industrial Technology (KITECH) 35-3 Hongchon-ri, Ipjang-myun, Seobuk-gu, Chonan-si, Chungnam 330-825, Republic of Korea; 6 Department of Chemical Engineering, Soongsil University, 511 Sangdo-dong, Seoul, Republic of Korea

## Abstract

Itaconate, a C_5_ unsaturated dicarboxylic acid, is an important chemical building block that is used in manufacturing high-value products, such as latex and superabsorbent polymers. Itaconate is produced by fermentation of sugars by the filamentous fungus *Aspergillus terreus*. However, fermentation by *A. terreus* involves a long fermentation period and the formation of various byproducts, resulting in high production costs. *E. coli* has been developed as an alternative for producing itaconate. However, fermentation of glucose gives low conversion yields and low productivity. Here, we report the whole-cell bioconversion of citrate to itaconate with enhanced aconitase and cis-aconitate decarboxylase activities by controlling the expression of multiple *cadA* genes. In addition, this bioconversion system does not require the use of buffers, which reduces the production cost and the byproducts released during purification. Using this whole-cell bioconversion system, we were able to catalyze the conversion of 319.8 mM of itaconate (41.6 g/L) from 500 mM citrate without any buffer system or additional cofactors, with 64.0% conversion in 19 h and a productivity of 2.19 g/L/h. Our bioconversion system suggests very high productivity for itaconate production.

Itaconate is a C_5_ unsaturated dicarboxylic acid that is produced from biomass and can be used in a number of high-value bio-based chemicals^1^. It is used industrially in the production of polymers such as synthetic latex, unsaturated polyester resins (UPR) and super absorbent polymers (SAP) and as a substitute for acrylic acid^2^. The total market demand for itaconate is increasing annually. In 2014, the global market for itaconate was valued at 126.4 million USD and is expected to increase to 204.6 million USD by 2023^3^.

Biosynthesis of itaconate using *Aspergillus itaconicus* was discovered by Kinoshita in 1931^4^. Since then, there have been many attempts to increase the titer of itaconate (Table 1). In 1945, Kane *et al*. reported itaconate production by *A. terreus*, which reached a titer of 27 g/L^5^. Apart from *Aspergillus* sp., other species have also been used to produce itaconate. Tabuchi *et al*. reported that *Candida* sp. can produce 35 g/L itaconate^6^. *Ustilago* sp. and *Pseudozyma* sp. were also shown to produce itaconate^7^,^8^,^9^. Currently, itaconate is produced using *A. terreus* with sugars as the substrate, and the titer and productivity reach 129 g/L and 1.15 g/L/h, respectively^10^. However, this titer is still lower than the expected theoretical maximum titer of 240 g/L by *A. niger*^11^, and the production cost is high due to high consumption of sugars, long duration of fermentation and purification of uncontrollable byproducts such as maleic acid, α-ketoglutaric acid, oxalic acid and other unidentified secondary metabolites^10^,^12^. Furthermore, the filamentous growth of fungi blocks the supply of oxygen during fermentation, which in turn hinders production of itaconate^13^. To address the oxygen supply issue and create a stable continuous system, bioconversion of citrate to itaconate using *A. terreus* in membrane bioreactor^14^, and fermentation using immobilized *A. terreus* has been also used for itaconate production^15^. However, the titer and productivity were uncompetitive compared to other conventional fermentation methods. In addition, *A. niger* has been developed as itaconate producer. However, the titer reached was only 26.2 g/L^16^. Although using a bacterial host for production has several advantages, such as rapid growth and easy controllability, *E. coli* does not have cis-aconitate decarboxylase (*cadA*), which converts cis-aconitate into itaconate. Since the discovery of CadA as the key enzyme in itaconate production^13^,^17^, strain of *E. coli* with heterologous expression of *cadA* gene was developed that can produce itaconate^11^. Vuoristo *et al*. produced itaconate using an *E. coli* strain expressing heterologous *cadA*, achieving a titer of 690 mg/L^18^. Another *E. coli* strain with random synonymous codon substitutions produced 7.23 g/L of itaconate^19^. Model-based metabolic engineering of *E. coli* increased itaconate production to 32 g/L^20^. However, *E. coli* fermentation is still not economically competitive with *A. terreus* fermentation in terms of titer and productivity. Thus, there is a need to develop additional strategies^20^.

Here, we suggest an efficient whole-cell bioconversion system for the production of itaconate instead of fermentation (Fig. 1). In our bioconversion system, citrate is converted into itaconate by recombinant *E. coli* cells expressing aconitase and cis-aconitate decarboxylase. Compared to conventional fermentation, this whole-cell bioconversion system can decrease the cost and time of process because *E. coli* is a fast-growing species and the conversion reaction is rapid. Additionally, bioconversion produces fewer byproducts, which is an advantage in the purification process. Furthermore, citrate, which is the substrate for our conversion system, is readily available. Therefore, we investigated the feasibility of a high-yield conversion process using an *E. coli* whole-cell biocatalyst and optimized the reaction conditions to establish an efficient and competitive itaconate production bioprocess that would improve yield and productivity.

## Results

### Construction of an *E. coli* whole-cell biocatalyst expressing *acn* and *cadA*

To construct the *E. coli* whole-cell biocatalyst, *acn* from *Corynebacterium glutamicum* ATCC 13032 and a synthetic, codon-optimized version of the *cadA* (cis-aconitate decarboxylase) gene of *A. terreus* were cloned into pCDF-duet1 (pHMS01). *acn* from *Corynebacterium glutamicum* ATCC 13032 was selected as it has the lowest K_m_ value to citrate among other prokaryotic species^21^. The *E. coli* strain BL21(DE3), which is typically used for protein production, was transformed with pHMS01. The expression of each gene was tested by running an SDS-PAGE and testing the whole-cell conversion reaction. Initially, BL21 containing pHMS01 converted only 3.4 mM of 100 mM citrate to itaconate (3.4%). SDS-PAGE analysis data showed that a portion of the CadA formed an inclusion body (data not shown). As shown in Fig. 2, changes in induction time and temperature influenced the expression of *cadA*. Conversion of citrate by BL21 containing pHMS01 increased 2.1-fold compared to the previous condition (7.2%). Although changing induction conditions increased the activities of the whole-cell, conversion was still low. Since CadA is the key enzyme for itaconate production^13^, the number of *cadA* gene copies was increased to overexpress cis-aconitate decarboxylase. The *cadA* gene was cloned into other duet vectors. pACYC-duet1 and pRSF-duet1 were tested for additional *cadA* expression. Increasing the number of *cadA* gene copies also improved the conversion of citrate. The addition of pHMS02 (pACYC-duet1::*cadA*) and pHMS03 (pRSF-duet1::*cadA*) (Fig. 2), each of which contained one *cadA* gene, improved citrate conversion compared to the pHMS01 single-vector system alone. The pHMS02 plasmid increased conversion from 7.2% to 10.2% and pHMS03 increased it to 15.9%. Furthermore, pHMS04 (pACYC-duet1::*cadA*::*cadA*) and pHMS05 (pRSF-duet1::*cadA*::*cadA*), which contain two *cadA* gene copies, improved conversion to a much greater degree. The pHMS04 plasmid increased conversion from 7.2% to 16.7%, while pHMS05 increased it to 25.7% (Fig. 2). All tested plasmids showed better conversion than the pHMS01 single-vector system. Comparing pHMS02 with pHMS03 and pHMS04 with pHMS05, pRSF-duet1 showed better expression than pACYC-duet1, as the former contains 100 copies and the latter fewer than 12 copies^22^. BL21 containing pHMS01 and pHMS05 (JY001) converted 25.7 mM of citrate to itaconate, resulting in a 7.6-fold increase which is equivalent to 25.7%. Thus, JY001 was used as a biocatalyst in further experiments.

### Selection of induction media and substrate concentration

To determine the optimal reaction conditions, several factors were evaluated. Induction medium and induction timing were selected to maximize the expression of *acn* and *cadA* and increase the mass of the biocatalyst. Since TB and 2x YT broth contained higher concentrations of nitrogen source and improved the yields of the plasmid over that of LB broth^23^, yields from those three media were compared to determine the highest protein expression and cell growth. Cells cultured in LB medium showed a higher production yield than cell cultured in the two other types of media. One milligram of whole-cell biocatalyst in LB broth converted 1.05 mM of citrate to itaconate per hour; the equivalent value for TB was 0.69 mM and for 2xYT, it was 0.52 mM (Fig. 3a). However, the dry cell weight (DCW) from TB was the highest, at 5.05 g/L, which was 7.2-fold higher than that of LB, and that of 2x YT was 1.71 g/L, which was 2.4-fold higher than that of LB (Fig. 3a). These properties conferred a longer and more stable growth following induction and showed the highest DCW in the TB medium^24^. Therefore, TB medium was selected as the induction medium, as it showed a higher conversion rate compared to the other two media tested (3.48 mM/h). Regarding the timing of induction, the addition of IPTG at the initial point of induction resulted in the highest production. Regarding the optimal citrate concentration, 81.9 mM itaconate was produced from 500 mM of citrate and conversion was 16.3% (Fig. 3b). Although conversion at this concentration was lower than at other concentrations, it produced the highest amount of itaconate compared to other citrate concentrations tested, and thus, 500 mM citrate was selected for use in further experiment.

### Effects of pH, temperature and permeability of whole cells

**Figure 1:**
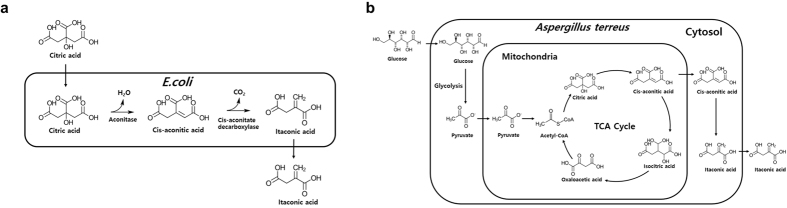
(**a**) *E. coli* whole-cell bioconversion strategy and (**b**) itaconate synthesis pathway in *A. terreus* (fermentation). In the *E. coli* whole-cell bioconversion strategy, two enzymes involved in this pathway, aconitase and cis-aconitate decarboxylase, were overexpressed. Unlike *A. terreus, E. coli* has no organelles, so the reaction proceeds spontaneously without the need for intracellular transport. In *A. terreus* fermentation, glucose is metabolized through glycolysis and the TCA cycle. Citric acid generated by the TCA cycle is converted to cis-aconitic acid by aconitase in the cytosol. Cis-aconitic acid is transferred to the mitochondria and converted to itaconic acid by cis-aconitate decarboxylase.

The optimal pH for Acn and CadA have been reported as 7.5–7.75 and 6.2, respectively^21^,^25^,^26^. However, the optimal pH for whole-cell conversion reaction was evaluated since overall reaction was mainly affected by initial extracellular pH in our previous study^27^. The optimal pH for the whole-cell reaction was pH 5.5, which is different from the optimal pH for each individual enzyme (Fig. 4a). The optimal temperatures for Acn and CadA were previously reported as 50 °C and 37 °C, respectively^21^,^25^,^26^. However, in our system, itaconate production was maximal at 35 °C (Fig. 4b). Under optimum pH and temperature, 239 mM itaconate was produced from 500 mM citrate (47.8%). Apart from pH and temperature, cofactors and FeSO_4_ also determined aconitase activity^28^. However, no significant effect was found in whole-cell system (Supplement Fig. 1). Fe^2+^ is a metal ion that positively influences aconitase activity^28^ but also inhibits cis-aconitate decarboxylase^29^. As a result, we did not observe any remarkable effects of FeSO_4_ on itaconate production.

**Figure 2:**
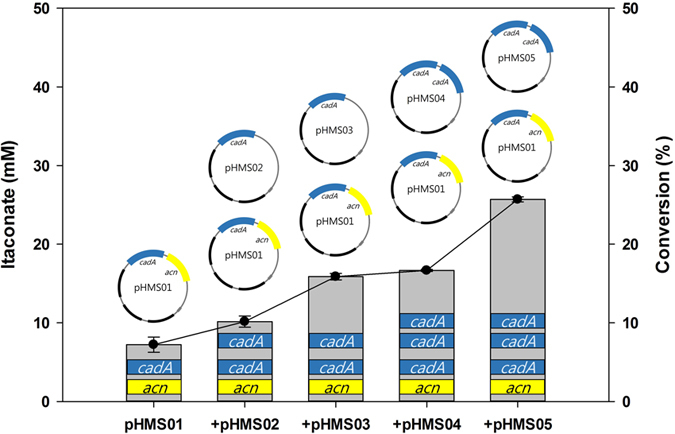
Conversion of citrate using a two vector system. Cells were grown in 5 mL of LB medium and 100 μM of IPTG for 48 h at 25 °C. The reaction was conducted using 100 mM citrate at pH 7 and 35 °C.

**Figure 3:**
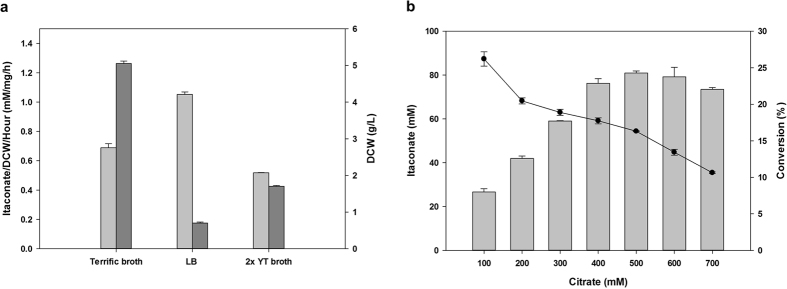
(**a**) Selection of induction medium. Enzyme expression was higher in LB. However, cell mass was higher in Terrific broth. Itaconate/DCW/Hour (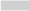); DCW (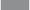). (**b**) Determination of the optimal citrate concentration; 500 mM citrate produced the highest amount of itaconate. The reaction was conducted at pH 7 and 35 °C for 24 h. Itaconate concentration (bar); Conversion (⚫).

Permeability is another factors that is important in the whole-cell reaction since the substrate and product have to be transferred through the cell membrane^30^. Rapid uptake of citrate and rapid excretion of itaconate would significantly increase the conversion rate. To increase *E. coli* membrane permeability, the citrate carrier protein and dicarboxylic acid transporter *citT* was overexpressed in JY002, and its effects on itaconate production were evaluated (Supplement Fig. 2); however, no significant increase in productivity was observed. Surfactant were also applied in an attempt to increase cell permeability (Supplement Fig. 3). There have been reports of using surfactants to increase production by whole-cell biocatalysts^31^,^32^. Two surfactants, polyoxyethylene sorbitan monooleate (Tween 80), which is a non-ionic surfactant, and sodium dodecyl sulfate (SDS) an anionic surfactant, were evaluated. SDS at a concentration of 0.01% had no significant effect, and >0.1% SDS resulted in no conversion activity due to cell lysis and protein destruction. In contrast, Tween 80 at a concentration of 0.1% resulted in a slight increase in conversion activity (Supplement Fig. 3). The optimum concentration of Tween 80 was found to be 0.5%, which resulted in the production of 260.5 mM itaconate (52.1%) (Fig. 5).

### Time-dependent monitoring of itaconate production

**Figure 4:**
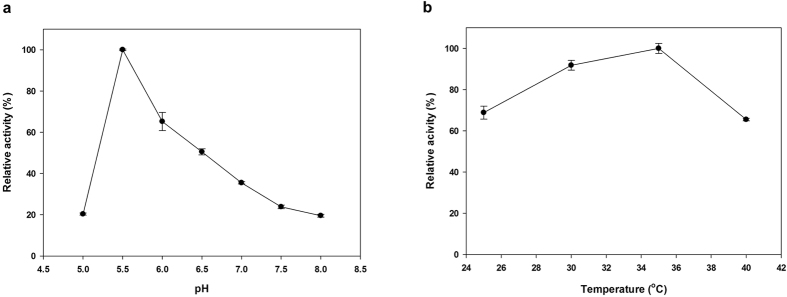
(**a**) Optimal pH and (**b**) temperature. A pH range from 5 to 8 and a temperature range from 25 to 40 °C was tested. A pH of 5.5 and a temperature of 35 °C were found to be optimal.

**Figure 5:**
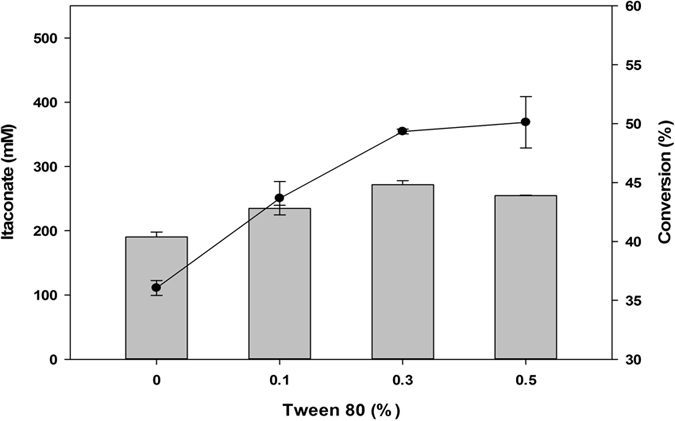
Effect of surfactant. Tween 80 increased conversion by 7.04% compared to the control. The reaction was conducted using 500 mM citrate, at pH 5.5 and 35 °C. Itaconate concentration (bar); Conversion (⚫).

**Figure 6:**
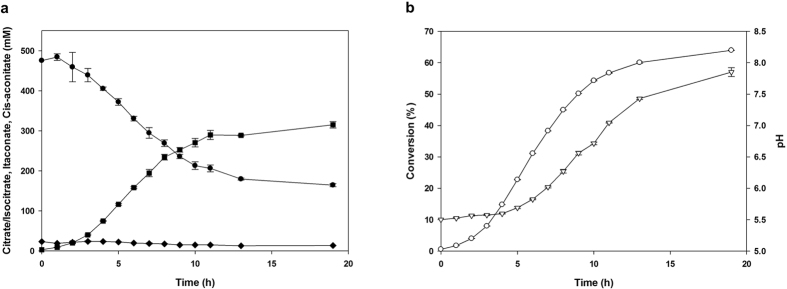
Time-dependent monitoring of itaconate production. Citrate/Isocitrate concentration (⚫); Itaconate concentration (■); Cis-aconitate concentration (♦); Conversion (○); and pH(▽).

We conducted itaconate production using the whole-cell conversion system under the optimum conditions outlined above (pH 5.5, 500 mM citrate, 0.5% Tween 80, JY001 cells at 35 °C). The conversion rate increased constantly until 6 h and decreased after 6 h. The reaction was almost saturated after 13 h and was completed at 19 h. The pH value also increased constantly to 7.9 in a manner similar to the conversion pattern (Fig. 6b). To control pH value constantly, buffers were applied. However, no significant effect was observed (Supplement Fig. 4). The concentration of cis-aconitate, an intermediate in the two-step reaction, increased in 3 h to 23.6 mM due to aconitase activity. After 3 h, the cis-aconitate level decreased steadily to 12.7 mM as it was converted to itaconate by cis-aconitate decarboxylase. After 19 h, there was no other byproduct produced and the efficiency of citrate conversion to itaconate was revealed as 100% since concentration of consumed citrate/isocitrate and produced itaconate was equal. At the end of the reaction, the itaconate concentration was 319.8 mM (41.6 g/L), equivalent to 64.0% conversion (Fig. 6a). Overall productivity was 2.19 g/L/h, and maximum productivity was 5.43 g/L/h within a 4 to 6 h period.

## Discussion

Many studies have reported improved itaconate production over several decades. Most recent studies have been based on a fermentation strategy using fungus *A. terreus* or *E. coli*. However, conventional fermentation requires long fermentation periods, high sugar consumption and unavoidable byproducts, which reduce productivity and increase production costs.

Therefore, we suggested bioconversion of itaconate from citrate using recombinant *E. coli* as a possible alternative method. Considering that *E. coli* is typically used for whole-cell reactions^32^,^33^ and that the speed of reaction by bioconversion is much faster than the conventional method (less than 19 h), resulting in high productivity, our bioconversion system could be comparable to conventional fermentation systems that use *A. terreus* or *E. coli*. Furthermore, our system uses citrate as the substrate, and the production of citrate is well established in the industry. *A. niger* produces 360 g/L of citric acid^34^, resulting in a cheap source of our starting material^35^,^36^.

To maximize the efficiency of our bioconversion system, we improved *acn* and *cadA* expression because aconitase and cis-aconitate decarboxylase is a critical enzyme for itaconate production^13^. Increasing *cadA* protein expression by increasing its gene copy number enhanced conversion yield by 18.5% (Fig. 2). However, increasing *acn* protein expression did not show significant effect on conversion (data not shown). This result shows that high expression of the *cadA* gene is more critical for this system. This is a reasonable proposition because aconitase and cis-aconitate decarboxylase compete for the key precursor, cis-aconitate (Fig. 1). Other studies have indicated the importance of *cadA* expression for itaconate production^19^,^20^. *E. coli* BL21(DE3) containing *acn* and three *cadA* genes yielded a much higher conversion (64.0%).

One of the important factors in itaconate production by bioconversion is the reaction conditions. Since itaconate production is the result of a two-step reaction, finding an optimal reaction condition was important. The optimal pH and temperature of the whole-cell biocatalyst were determined to be pH5.5 and 35 °C. The conversion was enhanced by adding 0.5% Tween 80, which increased the permeability of the cell membrane. The final concentration of itaconate obtained was 319.8 mM (41.6 g/L), a 64.0% conversion. This is still lower than that of other whole-cell reactions used in the production of other chemicals, such as cadaverine and acrylamide^27^,^37^. There are various possibilities on incomplete citrate conversion. It may be due to ceased utilization of citrate by pH or isocitrate production as a byproduct. However, considering that the overall productivity of a *A. terreus* fermentation is only 1.15 g/L/h^10^, the bioconversion of citrate to itaconate could be an alternative for itaconate production, as it shows an average productivity of 2.19 g/L/h and a maximum productivity of 5.43 g/L/h (4~6 h). This bioconversion system can be used in the production of itaconate without buffer as a whole-cell system. This represents a further advantage in that no purification is needed and this decreases the production cost.

In conclusion, we have developed the first *E. coli* whole-cell bioconversion system for the production of itaconate. The presence of multiple copies of *cadA* and optimization of the reaction conditions resulted in itaconate production at a level comparable to that achieved in traditional methods.

## Materials and Methods

### Reagents

Sodium citrate dihydrate (Dae-Jung, Korea) and citric acid (Duk-San Pure Chemical Co., Korea) were used to prepare a 1 M citrate solution. Citric acid, itaconic acid (Sigma-Aldrich, USA) and cis-aconitic acid (Alfa Aesar, USA) were used for in the preparation of a standard curve. PCR was performed with *n*Pfu-Forte polymerase (Enzynomics, Daejeon, Korea). Amplified DNA and plasmid were purified with MG Plasmid SV Miniprep Kit (Doctor Protein, Seoul, Korea). All endonucleases (*Nde*I, *EcoR*V, *BamH*I, *Sac*I, *Xho*I, and *Not*I) were purchased from New England Biolabs (USA).

### Bacterial strains, plasmids, primers and media

*E. coli* strains and plasmids used in this study are listed in Table 2. All the *E. coli* strains were pre-cultured in 5 mL Luria-Bertani (LB) medium (10 g/L tryptone, 5 g/L yeast extract and 5 g/L NaCl) containing appropriate antibiotics (kanamycin 50 μg/mL, spectinomycin 100 μg/mL, and chloramphenicol 25 μg/mL) for 16 h at 37 °C. A total of 2% of the pre-cultured cells were inoculated in 50 mL of induction medium. LB medium, Terrific Broth (TB) medium (12 g/L tryptone, 24 g/L yeast extract, 72 mM K_2_HPO_4_, 17 mM KH_2_PO_4_ and 0.4%v/v glycerol) and 2x YT Broth medium (16 g/L tryptone, 10 g/L yeast extract, 5 g/L NaCl) were compared as induction media. To maintain the plasmids, appropriate antibiotics were added to the media. Initially, the cells were induced under 30 °C for 24 h with 0.5 mM of isopropyl β-D-1-thiogalactopyranoside (IPTG) at initial point. To prevent the formation of enzyme inclusion bodies, the induction temperature and time were adjusted to 25 °C for 48 h. The induction culture was carried out in a 250 mL baffled Erlenmeyer flask with a shaking incubator (HB-201SL, Han-Baek, Korea) at 25 °C, 200 rpm.

### Genetic methods

A general molecular biology method was used for gene cloning^38^. Based on the sequence of cis-aconitate decarboxylase, originally from *A. terreus, cadA* was codon-optimized and synthesized by Cosmogenetech. (Seoul, Korea). The full sequence of the synthesized *cadA* gene is provided in the supplementary material. The *acn* gene encodes aconitase originally from *Corynebacterium glutamicum* ATCC 13032, and *citT* encodes a citrate carrier protein originally from *E. coli* K12. The *acn, cadA* and *citT* sequences were amplified by PCR using the primers listed in Table 2. The amplified DNA was purified and digested with endonucleases. *acn* was digested with *Nde*I and *EcoR*V, *cadA* at MCS1 with *BamH*I and *Sac*I, and at MCS2 with *Nde*I and *Xho*I, and *citT* was digested with *Sac*I and *Not*I. The digested PCR products were ligated to vectors that had been digested with the same endonuclease. Ligated plasmids were transformed into *E. coli* DH5α competent cells using the heat-shock method^39^. Constructed plasmids (Fig. 2) were confirmed using sequencing (Cosmogenetech, Seoul, Korea) and were used for further experiments.

The expression of each enzymes was tested by conversion reactions and SDS-PAGE (Sodium Dodecyl Sulfate PolyAcrylamide Gel Electrophoresis). A total of 1 mL of culture broth was centrifuged for cell harvest and washed with 50 mM of phosphate-buffed saline (PBS) pH 6.8. Harvested cells were treated with Bugbuster^TM^ (Novagen, Madison, WI, USA) under the conditions recommended by the manufacturer. After centrifugation at 15,184 × g for 3 min, the supernatant was sampled as the soluble fraction. The pellet of the insoluble fraction was washed 3 times and re-suspended in the same volume of PBS. SDS-PAGE was performed at 120 V for 2 h.

### Whole-cell reaction

The activity of Acn and CadA as single whole-cell biocatalysts was determined. Initially, the reaction was carried out at 35 °C and pH 7 for 24 h. For optimization, the total reaction volume was 500 μL, containing 500 mM citrate and 43.5 mg of cells^27^. For time-dependent monitoring of the conversion reaction, the total volume was 20 mL with optimized conditions, and the reaction solution was mixed intermittently for sampling every hour. The cover of the reactor was lifted whenever a sample was obtained. The reaction was stopped by heating at 90 °C for 5 mins. The reaction solution was then diluted at 1:50 and analyzed by HPLC. The optimal pH was determined using 1 M trisodium citrate dihydrate (pH 8.7) and 1 M citric acid (pH 1.5) stocks. Activity of each gene was examined by production of itaconate and presented in supplementary Table 2.

### Effect of additives on bioconversion reaction

FeSO_4_ as cofactor, and polyoxyethylene sorbitan monooleate (Tween 80) and Sodium Dodecyl Sulfate (SDS) (as cell membrane weakening agents) were tested. FeSO_4_ was prepared at a 10 mM concentration, and Tween 80 and SDS were used at 10%. Each reagent was added to the reaction solution. The final concentration of FeSO_4_ was 0, 5, 10, 15, or 20 μM, and those of Tween 80 and SDS were 0.01, 0.1, or 1%. Since Tween 80 caused increased conversion, the optimal concentrations of Tween 80 were tested from at concentrations ranging from 0.1 to 0.5%.

### Analytical methods

Citrate, isocitrate, itaconate, and cis-aconitate were quantified by high-performance liquid chromatography (HPLC). The reaction solution was heated to 90 °C for 5 min to stop the reaction and to aggregate proteins and then centrifuged at 15,314 × g to eliminate cell debris. The supernatant was diluted to 1:50 and filtered through a Whatman syringe filter (0.2 μm, PVDF, Sigma-Aldrich, St. Louis, USA). The filtrate was analyzed by HPLC (Prominence-i LC-2030, Shimadzu, Japan) equipped with an Aminex HPX-87H column (300 × 7.8 mm, 9 μm, Bio-Rad, Hercules, California, USA). The solution was eluted using 0.004 M H_2_SO_4_ as the mobile phase with flow rate of 0.6 mL/min flow rate. The oven temperature was set at 60 °C, and a UV detector was used to monitor the analytes at 210 nm^40^,^41^. Concentrations of each organic acid were calculated with standard curve and retention time of citrate, isocitrate, itaconate, and cis-aconitate were 7.6, 7.7, 12.0, and 6.8, respectively.

## Additional Information

**How to cite this article**: Kim, J. *et al*. Production of itaconate by whole-cell bioconversion of citrate mediated by expression of multiple cis-aconitate decarboxylase (*cadA*) genes in *Escherichia coli. Sci. Rep.* **7**, 39768; doi: 10.1038/srep39768 (2017).

**Publisher's note:** Springer Nature remains neutral with regard to jurisdictional claims in published maps and institutional affiliations.

## Supplementary Material

Supplementary Data

## Figures and Tables

**Table 1:** Development of itaconate production.

Strain	Initial substrate concentration	Titer	Productivity	Reference
*A. itaconicus*	Sucrose or Glucose 250 g/L	N/D	N/D	4
*A. terreus*	Molasses 98 g/L	27 g/L	0.08 g/L/h	5
*Candida sp.*	Glucose 100 g/L	35 g/L	0.28 g/L/h	6
*U. maydis*	Glucose 120 g/L	53 g/l	N/D	7
*Pseudozyma sp.*	Glucose or glycerol 80 g/L	16.7 g/L	0.11 g/L/h	9
*A. terreus*	Glucose 180 g/L	129 g/L	1.15 g/L/h	10
*A. niger*	Glucose 100 g/L	26.2 g/L	0.35 g/L/h	17
*E. coli*	Glucose 9.01 g/L	0.69 g/L	0.01 g/L/h	18
*E. coli*	Glycerol 70 g/L	7.23 g/L	0.08 g/L/h	19
*E. coli*	Glucose 27 g/l	32 g/L	0.45 g/L/h	20

N/D; No data.

**Table 2:** Strains, plasmids, and primers.

Strain, plasmid, and primer	Relevant information	Source or reference
Bacterial strains		
*E. coli* strains		
DH5α	F^*−*^ φ80*lacZ* M15 *endA recA hsdR*(r_k_^−^m_k_^−^) *supE thi gyrA relA* Δ(*lacZYA*-*argF*)U169	42
BL21(DE3)	F^*−*^ *ompT hsdS*_*B*_(r_B_^−^m_B_^−^) *gal dcm*	Novagen
JY001	BL21(DE3) carrying pHMS01 & pHMS05	(This study)
JY002	BL21(DE3) carrying pHMS01 & pHMS06	(This study)
Plasmids		
pACYC-duet1	Co-expression vector, Cm^r^. 2 MCS site with T7 promoter, lac operator, RBS. P15A replicon. 10 to 12 copy number.	Novagen
pCDF-duet1	Co-expression vector, Spec^r^. 2 MCS site with T7 promoter, lac operator, RBS. CloDF13 replicon. 20 to 40 copy number.	Novagen
pRSF-duet1	Co-expression vector, Km^r^. 2 MCS site with T7 promoter, lac operator, RBS. RSF1030 replicon(NTP1). Over 100 copy number.	Novagen
pHMS01	pCDF- duet1, Spec^r^, *cadA* (MCS1) and *acn* (MCS2).	(This study)
pHMS02	pACYC-duet1, Cm^r^, *cadA* (MCS1).	(This study)
pHMS03	pRSF-duet1, Km^r^, *cadA* (MCS1).	(This study)
pHMS04	pACYC-duet1, Cm^r^, 2 *cadA* (MCS1,2).	(This study)
pHMS05	pRSF-duet1, Km^r^, 2 *cadA* (MCS1,2).	(This study)
pHMS06	pRSF-duet1, Km^r^, 2 *cadA* (MCS1,2), *citT* (MCS1).	(This study)
Primers		
*acn* F	CTCTAT CATATG ATGGAGCTCACTGTGACTGA	
*acn* R	CTCT GATATC TTACTTAGAAGAAGCAGCCATC	
*cadA* F (MCS1)	CTCT GGATCC AATGACCAAGCAGAGCGCAG	
*cadA* R (MCS1)	CTCT GAGCTC TTAAACCAGCGGGGATTTAAC	
*cadA* F (MCS2)	ATCGTC CATATG ATGACCAAGACGAGCGCAG	
*cadA* R (MCS2)	CTCT CTCGAG TTAAACCAGCGGGGATTTAAC	
*citT* F	CTCT GAGCTC GCACTTGATAAATTTGGAAA	
*citT* R	CTCTCT GCGGCCGC TTAGTTCCACATGGCGAG	
