# Supplementary material for: Production of itaconate by whole-cell bioconversion of citrate mediated by expression of multiple cis-aconitate decarboxylase (cadA) genes in Escherichia coli
Source: Sci Rep. 2017 Jan 4;7:39768. doi: 10.1038/srep39768 (PMC5209708; doi:10.1038/srep39768)
Supplement: Supplementary Data [file srep39768-s1.pdf]

# Production of itaconate by whole-cell bioconversion of citrate mediated by expression of multiple cis-aconitate decarboxylase (*cadA*) genes in *Escherichia coli*

Junyoung Kim[1)], Hyung-Min Seo[1)], Shashi Kant Bhatia[2)], Hun-Seok Song[1)], Jung-Ho Kim[1)], Jong-Min Jeon[1)], Kwon Young Choi[3)], Wooseong Kim[4)], Jeong-Jun Yoon[5)], Yun-Gon Kim[6)], and Yung-Hun Yang [1)*]

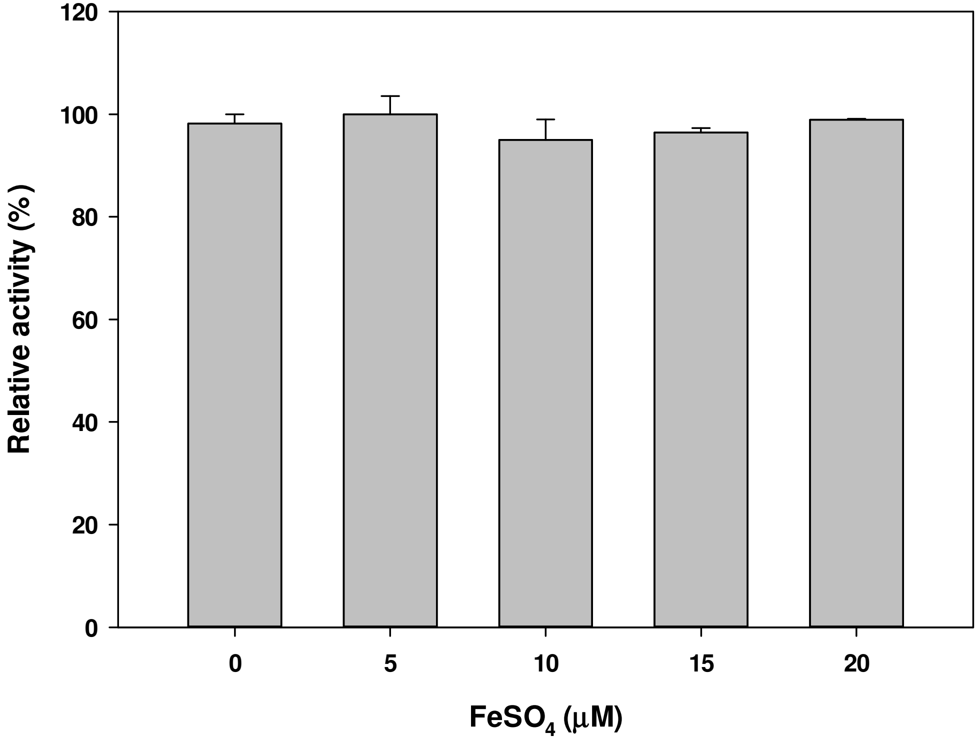


Supplement Figure 1. Effect of $FeSO_4$. No effect was detected.

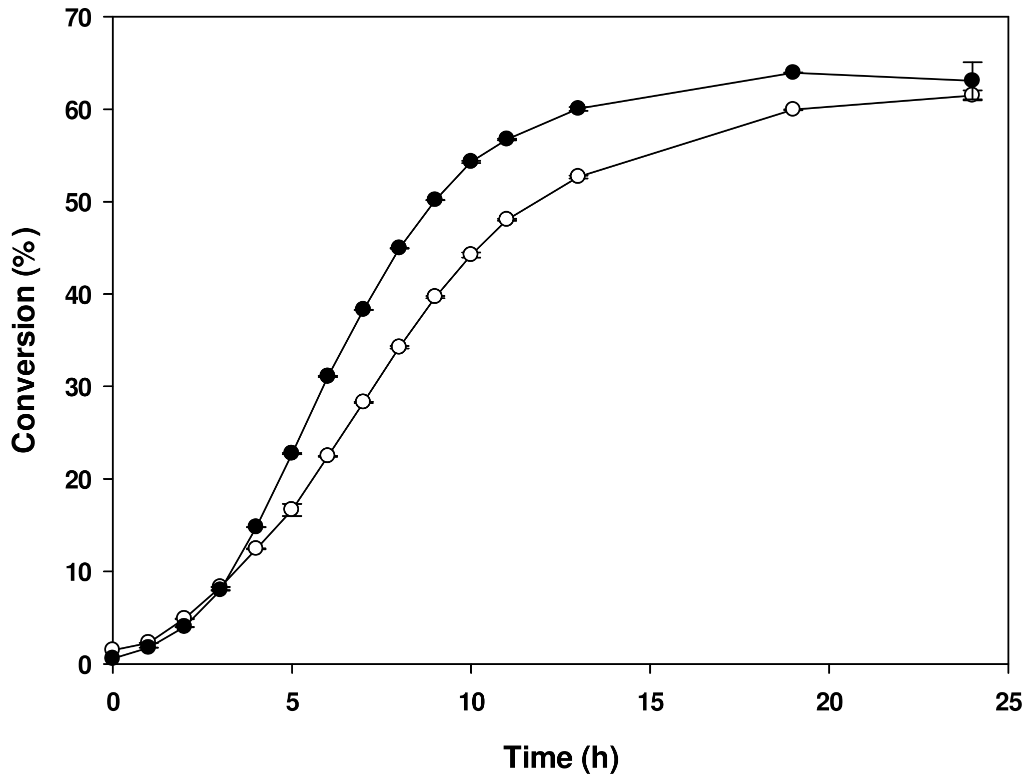


**Supplement Figure 2.** *citT*, a citrate carrier protein, had no effect on conversion. JY001 (●) and JY002 (○).

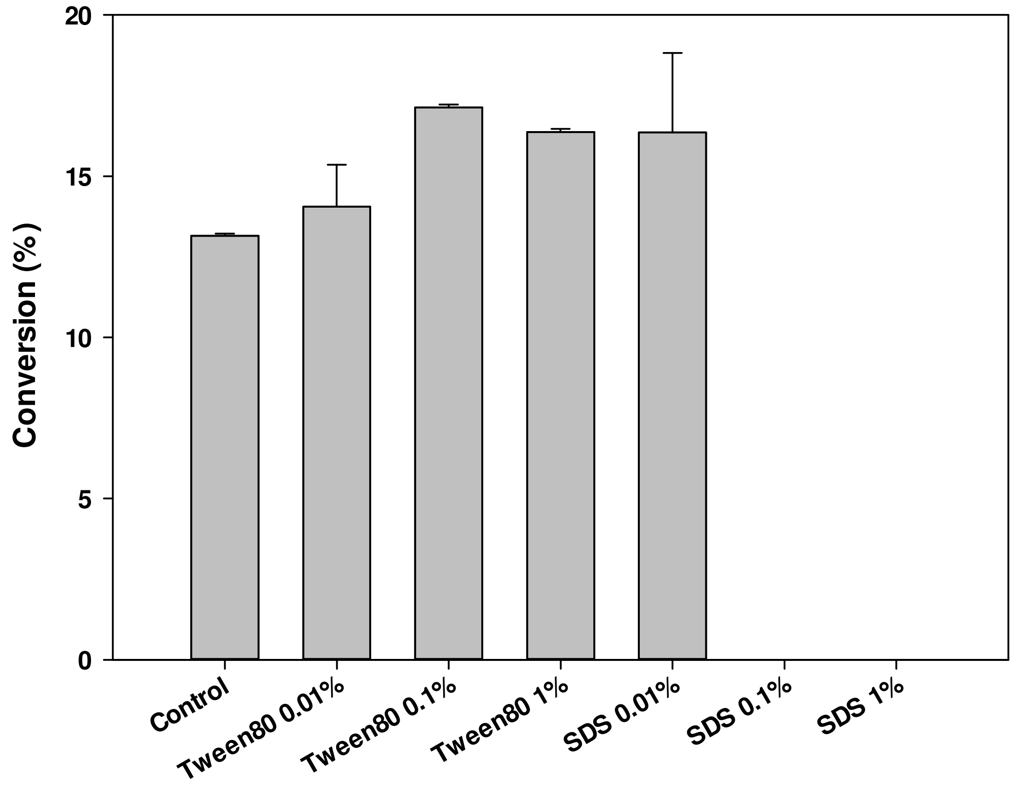


**Supplement Figure 3. Surfactant screening. Tween 80 and SDS were tested. The reaction was conducted at pH 7 and 35°C.**

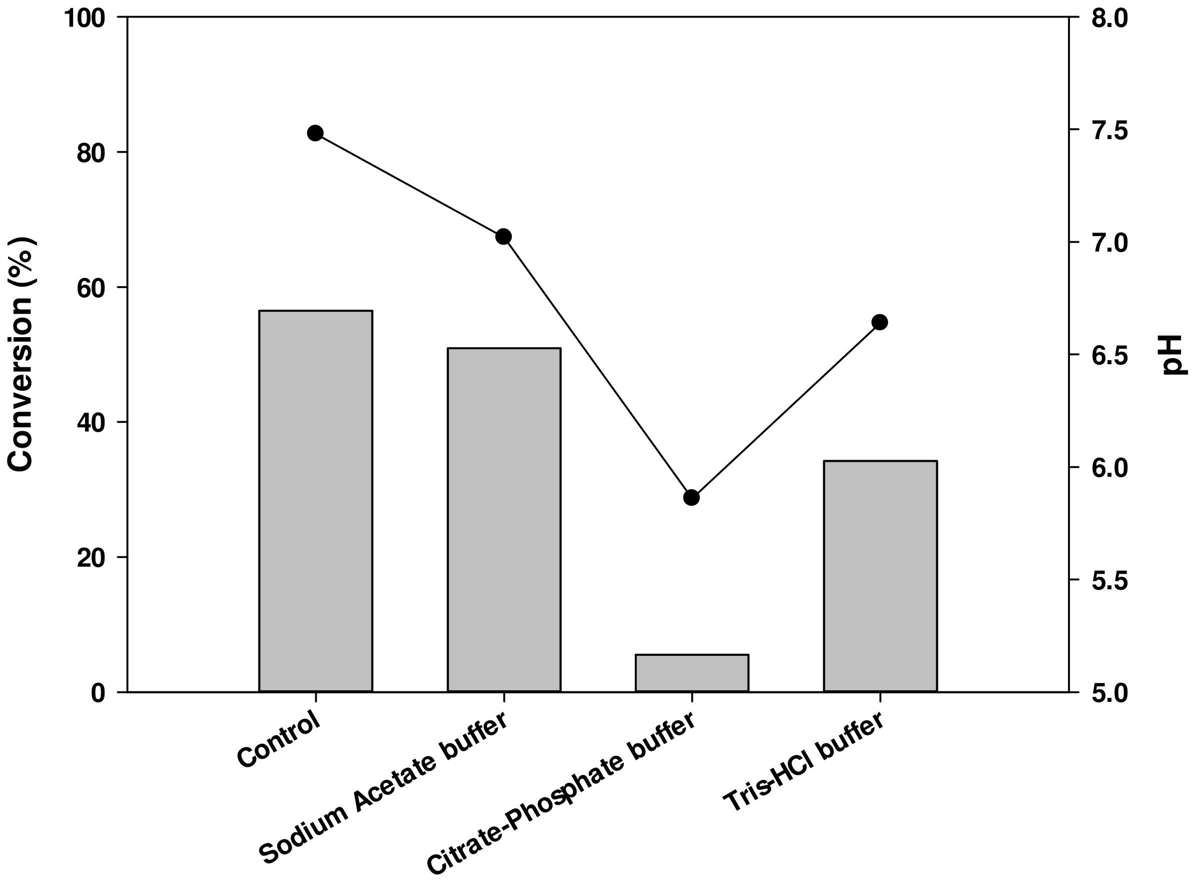


**Supplement Figure 4. Effect of Buffers. Sodium acetate buffer (pH 5.5, 300mM), Citrate-Phosphate buffer(pH 5.5, 300mM), Tris-HCl buffer(pH 6.8, 300mM) was tested. However, none of these showed positive effect.**

**Supplementary table 1. Activity of each enzymes**

| Strain | *acn* | *cadA* | Activity (U) | Relative activity |
|---|---|---|---|---|
| pCDF (*cadA*), pRSF (*cadA*x2) | X | OOO | 0.38 | 1 |
| pCDF (*acn*,*cadA*), pRSF (*cadA*x2) | O | OOO | 1.46 | 3.5 |

| Strain | *acn* | *cadA* | Activity (U) | Relative activity |
|---|---|---|---|---|
| pCDF (*acn*) | O | X | 0 | - |
| pCDF (*acn*,*cadA*) | O | O | 0.002 | 1 |
| pCDF (*acn*,*cadA*), pRSF (*cadA*) | O | OO | 0.151 | 75.5 |
| pCDF (*acn*,*cadA*), pRSF (*cadA*x2) | O | OOO | 1.46 | 730 |

Unit (U) = Amount of whole cell catalyst converting 1μmol substrate to 1μmol itaconate per minute.

## Supplementary table 2. DNA and amino acid sequences of synthetic *cadA*

| DNA sequence of synthetic cadA |
|---|
| ATGACCAAGCAGAGCGCAGATAGCAACGCAAAGAGCGGTGTCACG |
| AGCGAAATCTGCCATTGGGCAAGCAACCTGGCAACGGATGATATCC |
| CAAGCGACGTACTGGAACGTGCAAAGTACCTGATCCTGGACGGTAT |
| CGCATGCGCTTGGGTAGGTGCACGTGTCCCTTGGTCTGAAAAGTACG |
| TCCAGGCAACTATGTCCTTCGAGCCGCCTGGTGCTTGTCGTGTAATCG |
| GTTACGGCCAGAAACTGGGTCCTGTGGCAGCCGCCATGACTAACAG |
| CGCATTCATCCAGGCAACTGAGCTGGACGACTACCATAGCGAGGCT |
| CCACTGCATAGCGCTAGCATCGTCCTGCCAGCTGTTTTCGCTGCTTCT |
| GAGGTACTGGCTGAGCAAGGTAAAACCATCTCCGGTATCGACGTCAT |
| CCTGGCTGCTATCGTGGGTTTCGAGAGCGGTCCGCGTATCGGTAAAG |
| CTATCTACGGCAGCGACCTGCTGAACAACGGTTGGCATTGCGGTGCT |
| GTGTACGGTGCTCCGGCGGGTGCACTGGCGACTGGTAAACTGCTGG |
| GTCTGACTCCGGATTCTATGGAAGATGCCCTGGGTATTGCCTGTACTC |
| AAGCCTGTGGTCTGATGTCTGCGCAATATGGTGGTATGGTTAAACGT |
| GTGCAGCACGGTTTCGCGGCGCGTAATGGTCTGCTGGGTGGTCTGCT |
| GGCGCACGGCGGCTATGAAGCGATGAAAGGCGTTCTGGAACGTTCT |
| TATGGCGGCTTCCTGAAAATGTTCACCAAAGGCAATGGCCGTGAAC |
| CGCCGTATAAAGAAGAGGAGGTGGTGGCGGGCCTGGGCTCTTTCTG |
| GCACACGTTCACCATTCGTATTAAACTGTACGCGTGCTGTGGCCTGG |
| TTCACGGCCCGGTAGAAGCGATTGAAAACCTGCAGGGCCGTTACCC |
| GGAACTGCTGAATCGTGCCAACCTGTCTAACATCCGTCACGTTCACG |
| TTCAGCTGTCTACCGCCTCTAACTCTCACTGTGGCTGGATTCCGGAA |
| GAACGTCCGATCTCTTCCATCGCGGGCCAGATGTCTGTTGCGTACATC |
| CTGGCCGTACAGCTGGTTGATCAGCAGTGCCTGCTGTCTCAGTTTTC |
| TGAATTTGACGACAACCTGGAACGCCCGGAAGTGTGGGACCTGGCG |
| CGTAAAGTGACCTCTTCCCAGTCCGAAGAATTTGACCAGGACGGCA |
| ACTGCCTGTCCGCGGGCCGCGTACGCATTGAATTTAACGACGGCTCC |
| TCCATTACCGAATCCGTTGAAAAACCGCTGGGCGTTAAAGAACCGAT |
| GCCGAACGAACGCATCCTGCACAAATATCGCACCCTGGCGGGCTCC |
| GTTACCGATGAATCCCGCGTTAAAGAAATTGAAGATCTGGTGCTGG |
| GCCTGGATCGCCTGACCGATATTTCCCCGCTGCTGGAGCTGCTGAAC |
| TGCCCGGTTAAATCCCCGCTGGTT |

| Amino acid sequence of synthetic cadA |
|---|
| MTKQSADSNAKSGVTSEICHWASNLATDDIPSDVLERAKYLILDGIA |
| CAWVGARVPWSEKYVQATMSFEPPGACRVIGYGQKLGPVAAAMT |
| NSAFIQATELDDYHSEAPLHSASIVLPAVFAASEVLAEQGKTISGIDVIL |
| AAIVGFESGPRIGKAIYGSDLLNNGWHCGAVYGAPAGALATGKLLGL |
| TPDSMEDALGIACTQACGLMSAQYGGMVKRVQHGFAARNGLLGGL |
| LAHGGYEAMKGVLERSYGGFLKMFTKGNGREPPYKEEEVVAGLGSF |
| WHTFTIRIKLYACCGLVHGPVEAIENLQGRYPELLNRANLSNIRHVHV |
| QLSTASNSHCGWIPEERPISSIAGQMSVAYILAVQLVDQQCLLSQFSE |
| FDDNLERPEVWDLARKVTSSQSEEFDQDGNCLSAGRVRIEFNDGSSI |
| TESVEKPLGVKEPMPNERILHKYRTLAGSVTDESRVKEIEDLVLGLDRL |
| TDISPLLELLNCPVKSPLV |
